# Paternal nicotine exposure defines different behavior in subsequent generation via hyper-methylation of *mmu-miR-15b*

**DOI:** 10.1038/s41598-017-07920-3

**Published:** 2017-08-04

**Authors:** Jingbo Dai, Zhaoxia Wang, Wangjie Xu, Meixing Zhang, Zijue Zhu, Xianglong Zhao, Dong Zhang, Dongsheng Nie, Lianyun Wang, Zhongdong Qiao

**Affiliations:** 1 School of Life Science and Biotechnology, Shanghai Jiao Tong University, 800 Dongchuan Road, Shanghai, 200240 P. R. China; 2 Bio-X Institutes, Key Laboratory for the Genetics of Developmental and Neuropsychiatric Disorders (Ministry of Education), Shanghai Jiao Tong University, 1954 Huashan Road, Shanghai, 200030 China; 3 Brain Science and Technology Research Centre, Shanghai Jiao Tong University, 800 Dongchuan Road, Shanghai, 200240 China; 4 Shanghai Key Laboratory of Psychotic Disorders, Shanghai Institute of Mental Health, Shanghai Jiao Tong University, 600 South Wan Ping Road, Shanghai, 200030 China; 5 Shanghai Key Laboratory of reproductive medicine, School of medicine, Shanghai Jiao Tong University, 280 South Chongqing Road, Shanghai, 200025 China; 6 College of Medicine, University of Illinois at Chicago, 909 S Wolcott Ave, Chicago, IL 60612 USA

## Abstract

The neurobehavioral effects of paternal smoking and nicotine use have not been widely reported. In the present study, nicotine exposure induced depression in the paternal generation, but reduced depression and promoted hyperactivity in F1 offspring. While this intergenerational effect was not passed down to the F2 generation. Further studies revealed that nicotine induced the down-regulation of mmu-miR-15b expression due to hyper-methylation in the CpG island shore region of *mmu-miR-15b* in both the spermatozoa of F0 mice and the brains of F1 mice. As the target gene of mmu-miR-15b, *Wnt4* expression was elevated in the thalamus of F1 mice due to the inheritance of DNA methylation patterns from the paternal generation. Furthermore, the increased expression of *Wnt4* elevated the phosphorylation level of its downstream protein GSK-3 through the canonical WNT4 pathway which involved in the behavioral alterations observed in F1 mice. Moreover, *in vivo* stereotaxic brain injections were used to induce the overexpression of mmu-miR-15b and WNT4 and confirm the neurobehavioral effects *in vitro*. The behavioral phenotype of the F1 mice resulting from paternal nicotine exposure could be attenuated by viral manipulation of mmu-miR-15b in the thalamus.

## Introduction

Tobacco smoking is one of the most severe public health issues worldwide^1^ and it has recently been associated with mental disorders, such as depression, attention-deficit hyperactivity disorder (ADHD), among others. In the smoking population, the lifetime prevalence of major depressive disorder (MDD) appears to be markedly higher, with rates as high as 53% (compared to 6% to 10% in non-smokers)^2^. From another perspective, patients suffering from depression are much more likely to smoke tobacco than those who do not have an underlying depressive disorder^3, 4^. These epidemiological data strongly support the high comorbidity of smoking with depressive symptoms because depression increases the risk of smoking and smoking increases the risk of depression. As one of the most hazardous substances in tobacco, nicotine plays a pivotal role in tobacco addiction^5^. As a consequence, there is an urgent need for additional research concerning the effects of nicotine on the nervous system because of the increasing popularity of E-cigarettes^6, 7^. There were very limited studies in both humans and animals have indicated that nicotine treatment affects several aspects of emotionality, but the exact effects remain controversial^8, 9^. Most mental disorders, including MDD and ADHD, are considered complex familial aggregative or heritable traits^10^. Therefore, it is worth considering whether the neuropsychiatric effects of smoking or nicotine exposure can be passed down to future generations. Most studies have focused on the effects of prenatal smoking or nicotine exposure on the mental health of the progeny^11–16^. ADHD is a developmental neuropsychiatric disorder, whose pathogenesis may be influenced by multiple environmental factors, including smoking and alcohol, and chemicals such as lead and polychlorinated biphenyls^17^. A limited number of studies have reported that paternal smoking influences the health of the next generation^18^, but the effects on the mental health of offspring have not been widely reported. However, because of the high comorbidity of paternal smoking with maternal passive smoking, the intergenerational effects of paternal smoking *per se* is difficult to observe in the population; consequently, the establishment of a paternal smoking animal model is necessary and important.

Epigenetic studies on neuropsychiatric disorders have shown that environmental factors are also critical and that the genetic make-up of an individual contributes to heritability and disease risk. Theoretically, inherited non-genetic changes might represent evolutionary responses that enable rapid adaptation to environmental variation. Interestingly, a previous study reported that people traumatized during the genocide of the Khmer Rouge tended to have children who suffered from anxiety and depression^19^; this effect might be attributable to small non-coding RNAs (sncRNAs) in sperm, which can transmit acquired traits to progeny^20^. Other epigenetic modifications, such as DNA methylation and histone modification, could also contribute to the passing of non-genetic changes to future generations^21–23^. These findings provide potential mechanisms for the intergenerational effects of paternal smoking or nicotine exposure. However, despite these advances, the current understanding of the father-to-offspring transmission of nicotine-induced behavioral changes remains unclear.

In the present study, we present an animal model of paternal smoking and nicotine exposure. In this model, sexually mature mice are exposed daily to tobacco smoke or intraperitoneal injections of nicotine. Exposure occurs throughout spermatogenesis to determine whether tobacco smoke or nicotine induce filial generational behavioral alterations. Through these efforts, we hope to characterize the molecular mechanisms underlying this putative intergenerational transmission process.

## Results

### Tobacco smoke exposure induced behavioral alternations in the treated mice and their progeny

**Figure 1:**
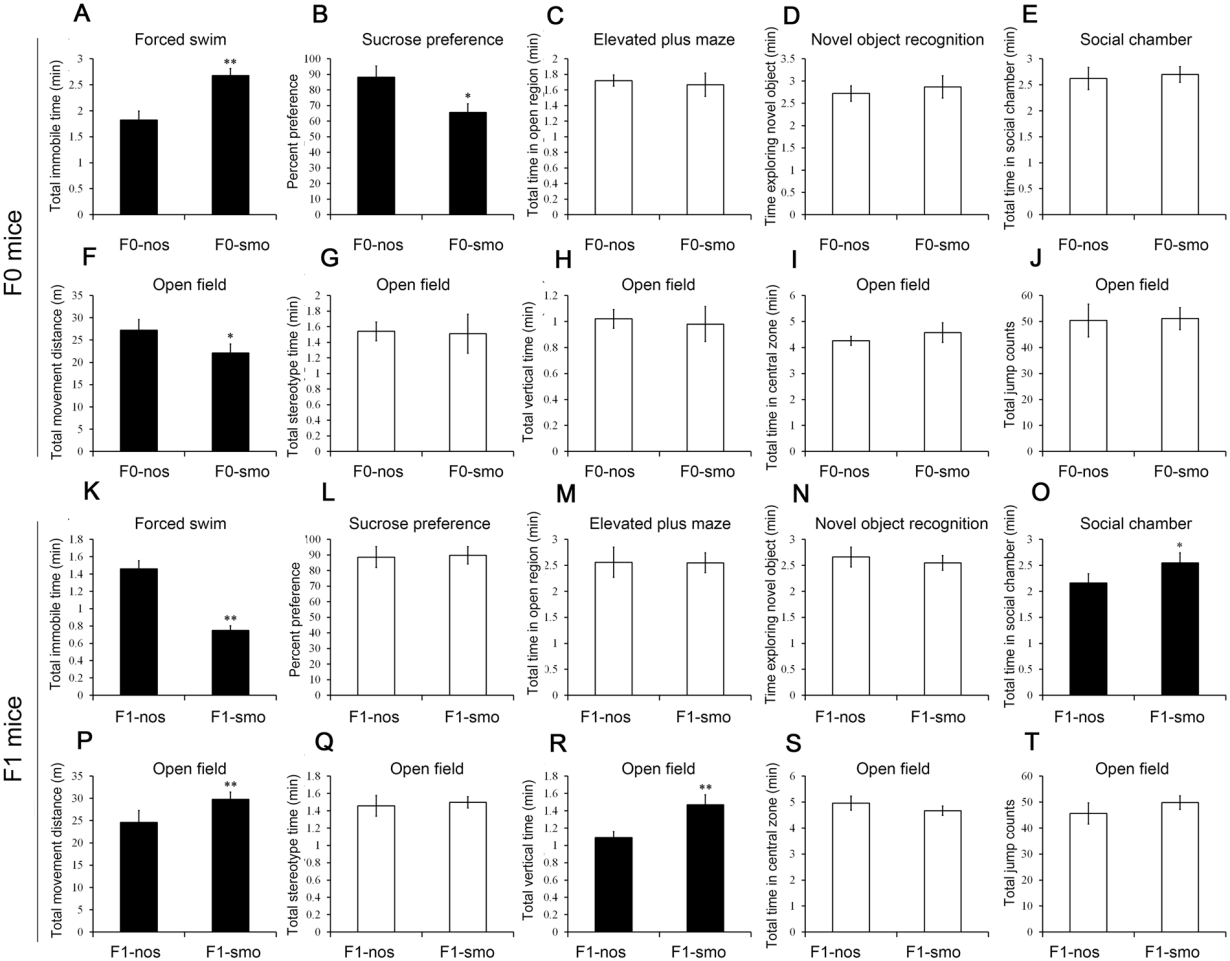
Behavioral tests of F0 and F1 mice of the tobacco smoke-treated and control groups (n = 20 for each group). (**A**) Forced swim tests of F0 mice from the tobacco smoke-treated and control groups. The histogram shows the total immobile times of the tested mice. (**B**) Sucrose preference test of F0 mice from the nicotine-treated and control groups. (**C**) Elevated plus maze test of F0 mice from the treated and control groups. (**D**) Novel object recognition test of F0 mice from the treated and control groups. (**E**) Social chamber test of F0 mice from the treated and control groups. (**F**–**J**) Open-field test of F0 mice from the treated and control groups. (**K**) Forced swim test of F1 mice from the paternal tobacco smoke-exposure and control groups. (**L**) Sucrose preference test of F1 mice from the paternal tobacco smoke-exposure and control groups. (**M**) Elevated plus maze test of F1 mice from the paternal tobacco smoke-exposure and control groups. (**N**) Novel object recognition test of F1 mice from the paternal tobacco smoke-exposure and control group. (**O**) Social chamber test of F1 mice from the paternal tobacco smoke-exposure and control groups. (**P**–**T**) Open-field test results for F1 mice from the paternal tobacco smoke-exposure and control groups.

F0 C57 male mice were subjected to tobacco smoke administration for 5 weeks to mimic long-term heavy smokers followed by behavioral tests to assess the neurobehavioral status of the mice. The tobacco smoke-treated mice (F0-smo) exhibited significantly longer immobility times (Fig. 1A, P = 0.001) in the forced swim test and reduced sucrose preferences (Fig. 1B, P = 0.028) compared with the non-smoking controls (F0-nos). In the open field test, F0-smo mice moved significantly shorter distances (Fig. 1F, P = 0.037). These results indicated that the F0-smo mice in this animal model exhibited depression and a hypo-activity phenotype. In other behavioral tests, including elevated plus maze (Fig. 1C), novel object recognition (Fig. 1D), social chamber (Fig. 1E) and open field tests (Fig. 1G–J), no significant differences between the two groups were detected.

The F1-nos and F1-smo offspring were obtained, and subsequently, animal behavioral tests were conducted. In the forced swim test, the total time of immobility was significantly reduced in the paternal tobacco smoke-treated group compared with the controls (Fig. 1K, P = 0.001). In the social chamber test, F1-smo mice exhibited a greater length of time in the social chamber (Fig. 1O, P = 0.042). In the open field test, the total distance moved (Fig. 1P, p = 0.009) and total vertical time (Fig. 1R, p = 0.001) of the F1-smo group were significantly elevated compared with the F1-nos group, while other parameters, including the total time of stereotypic behavior (Fig. 1Q), the total time in the central zone (Fig. 1S) and jump count (Fig. 1T), were not significantly different between the two groups. No significant differences between the two groups were observed in the sucrose preference (Fig. 1L), elevated plus maze test (Fig. 1M) or novel object recognition test (Fig. 1N). These results suggest that tobacco smoke exposure might induce a depressive phenotype in the F0 generation, resulting in hyperactivity and activated social behavior in the F1 generation.

### Nicotine exposure induced behavioral alterations in the treated mice and their progeny similar to that induced by tobacco smoke

**Figure 2:**
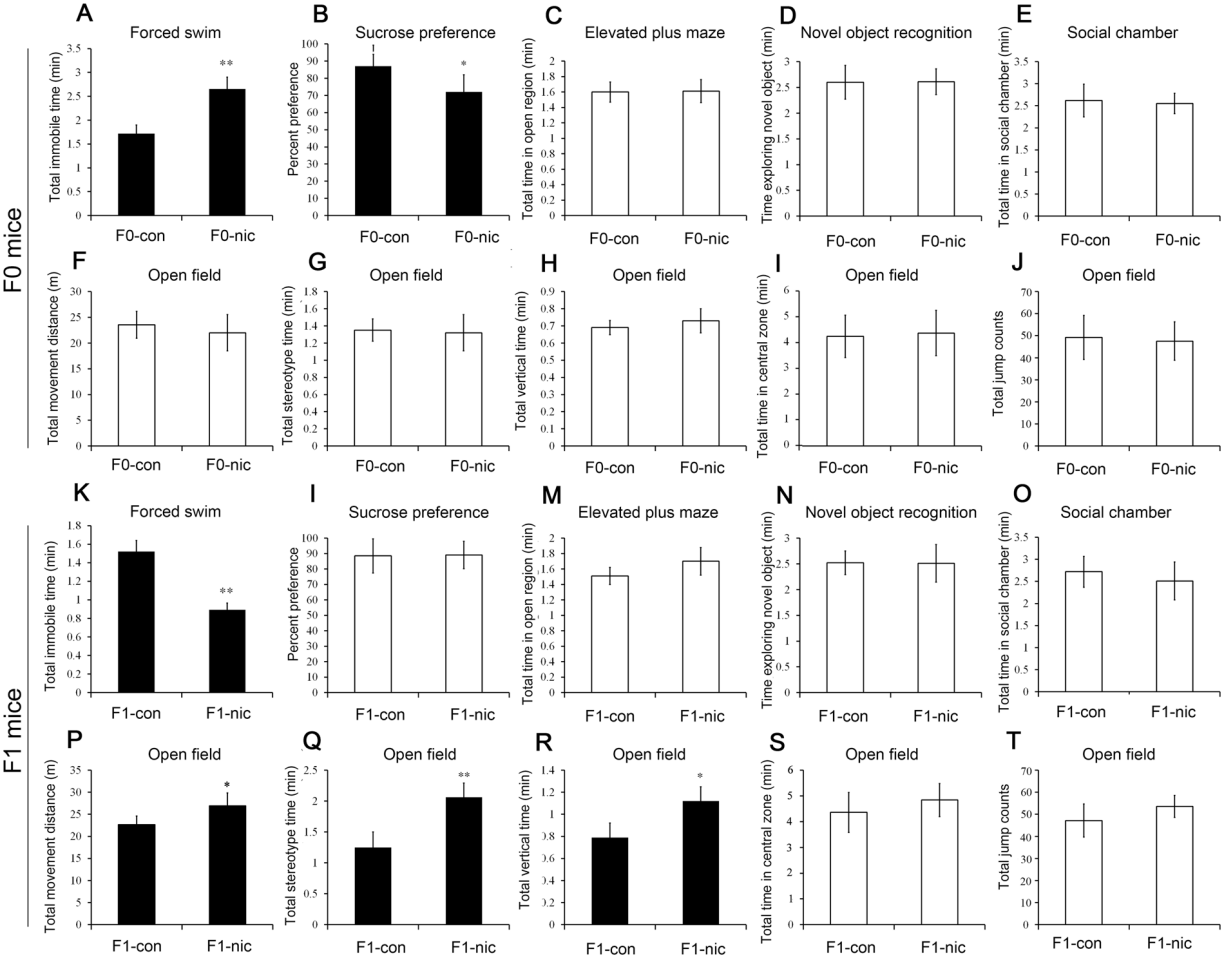
Behavioral tests of F0 and F1 mice of the nicotine-treated and control groups (n = 20 for each group). (**A**) Forced swim tests of F0 mice from the nicotine-treated and control groups. (**B**) Sucrose preference test of F0 mice from the nicotine-treated and control groups. (**C**) Elevated plus maze test of F0 mice from the nicotine-treated and control groups. (**D**) Novel object recognition test of F0 mice from the nicotine-treated and control groups. (**E**) Social chamber test of F0 mice from the nicotine-treated and control groups. (**F**–**J**) Open-field test of F0 mice from the nicotine-treated and control groups. (**K**) Forced swim test of F1 mice from the paternal nicotine-exposure and control groups. (**L**) Sucrose preference test of F1 mice from the paternal nicotine-exposure and control groups. (**M**) Elevated plus maze test of F1 mice from the paternal nicotine-exposure and control groups. (**N**) Novel object recognition test of F1 mice from the paternal nicotine-exposure and control group. (**O**) Social chamber test of F1 mice from the paternal nicotine-exposure and control groups. (**P**–**T**) Open-field test results for F1 mice from the paternal-nicotine exposure and control groups.

After administration of this compound for 5 weeks, the neurobehavioral status of the mice was evaluated. The nicotine-treated mice (F0-nic) exhibited significantly longer immobility times (Fig. 2A, P = 0.003) in the forced swim test and a reduction in sucrose preference (Fig. 2B, P = 0.030) relative to controls (F0-con). These results suggested that the F0 mice in this model exhibited a depression-like phenotype. In the other behavioral tests (Fig. 2C–J, no significant differences between the two groups were detected.

The F1-con and F1-nic offspring were obtained, and animal behavioral tests were conducted at 8–10 weeks of age. In the forced swim test, the total time of immobility was significantly shortened in the paternal nicotine-treated group compared with the controls (Fig. 2K, P = 0.001). In the open field test, the total distance moved (Fig. 2P, p = 0.037), total time of stereotypic behavior (Fig. 2Q, p = 0.007) and total vertical time (Fig. 2R, p = 0.019) of the F1-nic group were significantly elevated compared with the F1-con group, while other parameters (Fig. 2S,T) were not significantly different. Additionally, no significant differences between the two groups were observed in other behavioral tests (Fig. 2L–O). These results suggested that paternal nicotine exposure led to decreased levels of depression-like behavior and increased activity in the F1 offspring. Thus, the behavioral tests revealed that paternal nicotine exposure might induce a depressive phenotype in the F0 generation, while attenuating depression and inducing hyperactivity in the F1 generation.

### Nicotine elevated the level of Wnt4 mRNA in the mouse spermatozoa

**Figure 3:**
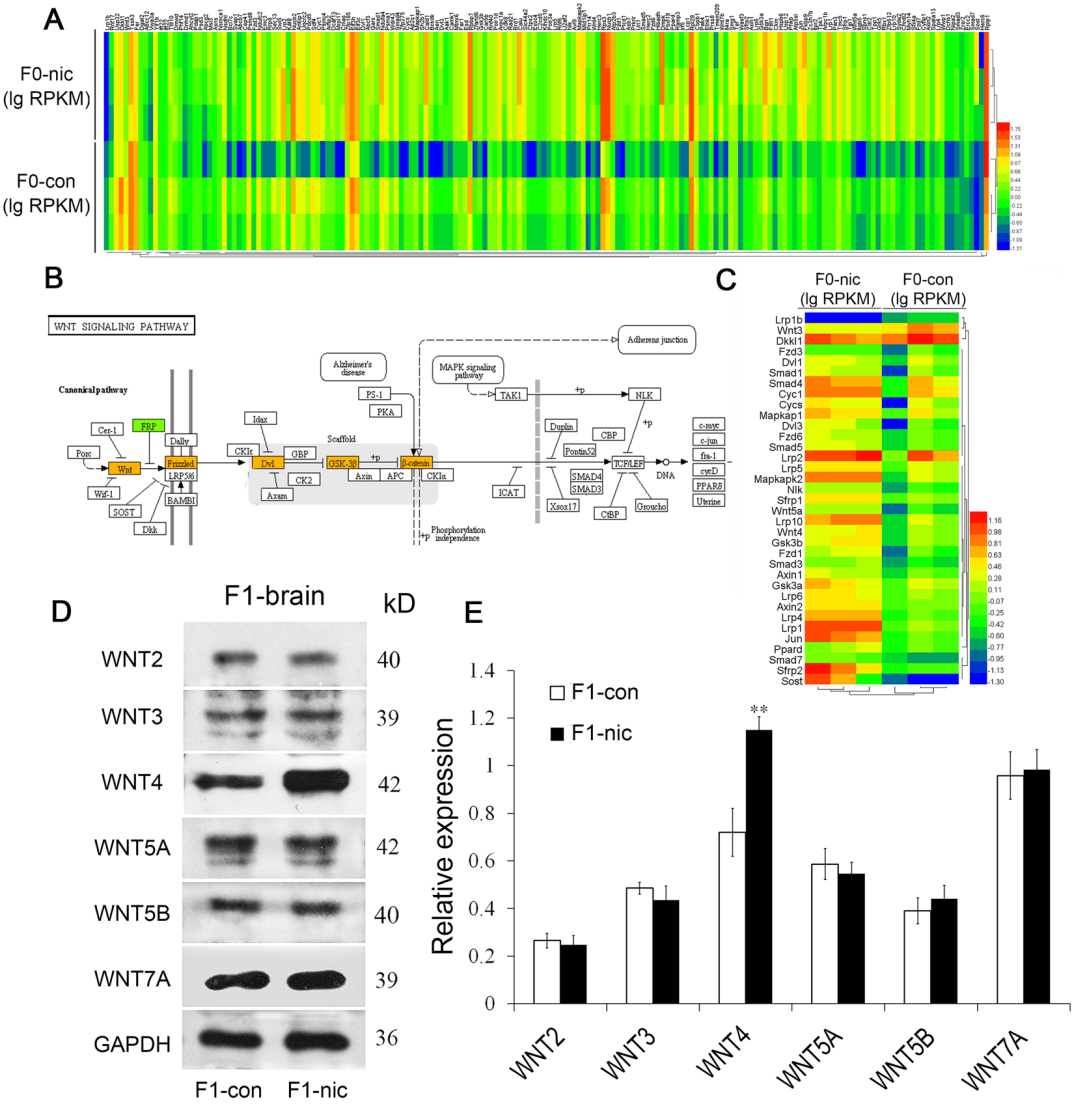
Transcriptomics analyses of the spermatozoa from nicotine-treated and control mice. (**A**) Heat map of Wnt/beta-catenin signaling-related mRNAs in the spermatozoa from nicotine-treated and control mice. The expression levels of mRNAs were normalized to lgRPKM prior to the transcriptomics analysis. (**B**) Schematic diagram of the canonical WNT signaling pathway (n = 3). (**C**) Heat map of key mRNAs in the Wnt signaling pathway in the spermatozoa of nicotine-treated and control mice. (**D**) Western blot results for WNT2, WNT3, WNT4, WNT5A, WNT5B and WNT7A from F1 brain tissue from the nicotine-treated and control groups. (**E**) A histogram of the western blot results for WNT2, WNT3, WNT4, WNT5A, WNT5B and WNT7A from F1 brain tissue (n = 3).

To acquire the gene transcription profiles of the sperm from the control and nicotine-treated F0 mice, murine sperm were purified (SFig. 1A), followed by the whole transcriptome sequencing, and the reads were mapped and analyzed. Figure 2A displays a heat map of the genes in the sperm differentially expressed resulting from nicotine treatment. The NGS results were subsequently validated using real-time PCR, which confirmed these alterations (SFig. 1B). The discrepancies correlated with the neurobehavioral differences between the two groups were intensively scrutinized. Additional bioinformatic analyses revealed that the nicotine-induced alterations primarily involved the enrichment of the expression of genes in the Wnt signaling pathway (Fig. 3B); the key genes *Wnt4*, *Fzd9*, *Dvl2* and *Gsk3* were all significantly up-regulated in nicotine-treated mice (Fig. 3C). In a previous study^24^, we demonstrated that only Wnt4 mRNA, but not WNT4 protein, could be detected in murine spermatozoa; accordingly, we only investigated the protein expression of Wnt family members in the brain tissue of F1 mice. The results revealed that only Wnt4 was significantly elevated in the F1-nic compared with the F1-con group, and other Wnt members (Fig. 2D and E) were not affected.

### Paternal nicotine exposure induced activation of the Wnt4 pathway in F1 mouse brain

**Figure 4:**
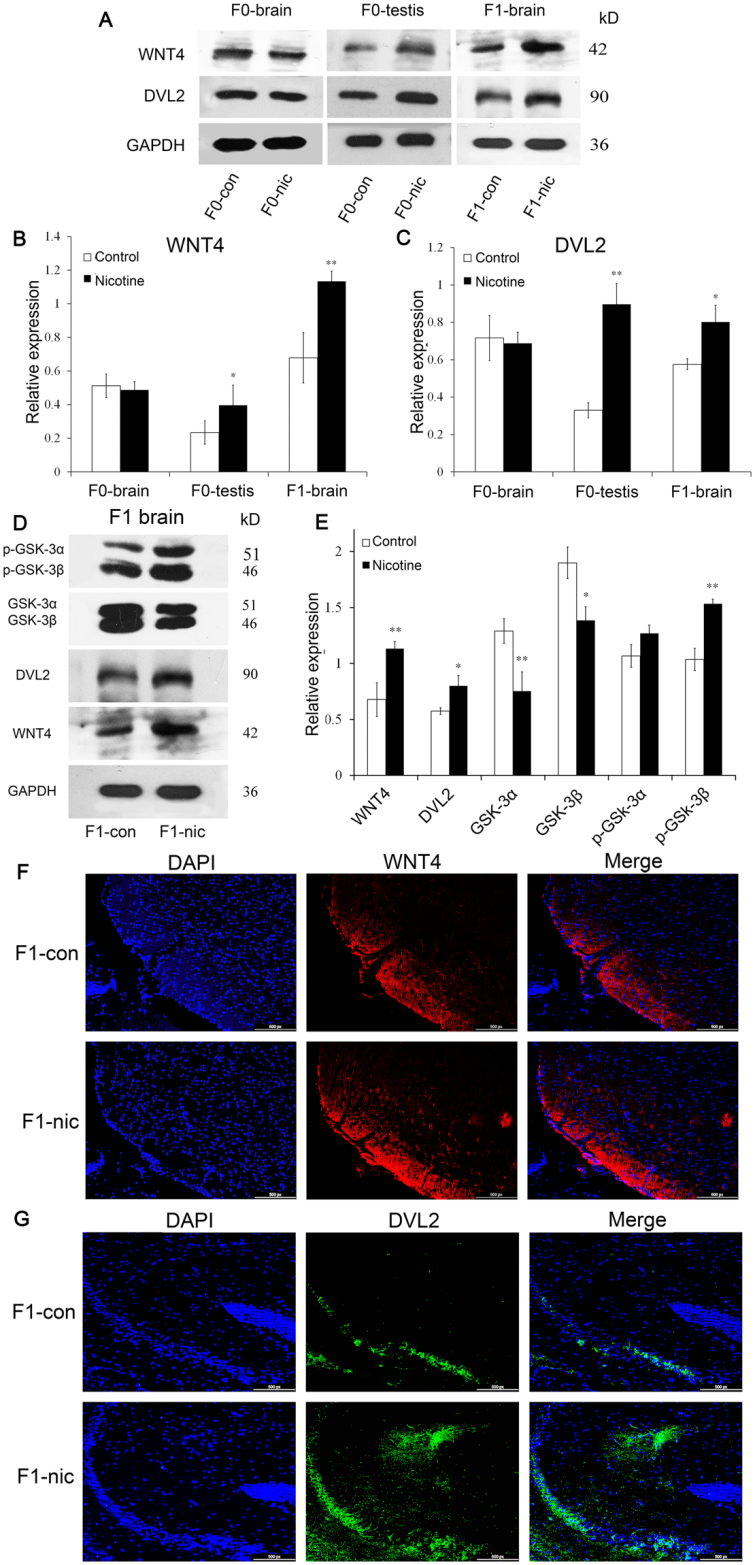
Expression levels and distribution of key proteins of Wnt4 signaling in different tissues. (**A**) Western blot results for WNT4 and DVL2 in F0-brain, F0-testis and F1-brain tissue of the two groups. (**B**) A histogram of the western blot results for WNT4 (n = 3). (**C**) A histogram of the western blot results for DVL2 (n = 3). (**D**) Western blot films of WNT4, DVL2 and GSK3 from F1-brain tissue from the nicotine-treated and control groups. (**E**) A histogram of the western blot results for WNT4, DVL2 and GSK3 in F1-brain tissue (n = 3). (**F**) Immunofluorescence staining for WNT4 (red) and DNA (blue) of brain samples from F1-con and F1-nic mice. (**G**) Immunofluorescence staining for DVL2 (green) and DNA (blue) in brain samples obtained from F1-con and F1-nic mice.

As the mRNA sequencing results suggested that the Wnt4 pathway might play an important role in the intergenerational effects of nicotine, the expression of two key proteins in Wnt4 signaling, WNT4 and Dishevelled 2 (DVL2) were measured using Western blot analysis (Fig. 3A) in the brain and testis tissues of F0 and F1 mice. The corresponding histograms for WNT4 (Fig. 3B) and DVL2 (Fig. 4C) revealed that nicotine elevated the expression of both proteins in the F0 testis (WNT4: P = 0.036, DVL2: P = 0.001) and the F1-brains (WNT4: P = 0.003, DVL2: P = 0.012), but not in the F0-brains. In the F1 mouse brains, the activation of Wnt4 signaling, which is closely associated with neurobehavioral status, was further investigated. Within the Wnt4 pathway, Wnt4 induces the normal inactivation of glycogen synthase kinase 3 (GSK3), evidenced by the stabilization of β-catenin and the stimulation of downstream gene transcription. Western-blot analysis (Fig. 4D and E) indicated that the expression of GSK-3α and -3β was significantly down-regulated, while phosphorylation (p-GSK3α/β) was elevated after nicotine treatment. For the mRNA level, *Wnt4* were elevated in both the sperm of F0-nic mice and the brain of F1-nic mice while the *Dvl2* was only found elevated in brain of F1-nic mice (SFig. 2A and B).

We determined the distribution of WNT4 in the F1-nic mouse brains via immunofluorescence. As shown in Fig. 4F, WNT4 was primarily expressed in the thalamus (TH), and the expression level of WNT4 in the brains of F1-nic mice was greater than that seen in the controls. Because it is a downstream target of activated Wnt4, the distribution of DVL2 was also assessed. DVL2 was highly expressed in the hippocampal formation (HPF). Figure 4G illustrates that DVL2 was significantly expressed in the HPF, particularly in the CA3 region, but not in the dentate gyrus (DG) (SFig. 2C), in the F1-nic brain. Paternal nicotine exposure was also found to elevate DVL2 expression in the CA3 region of F1-nic mice. Thus, we speculated that in the F1 offspring following paternal nicotine exposure, the expression of WNT4 would be up-regulated in the TH, and the Wnt4 pathway would be activated in the HPF via a paracrine manner.

### Mmu-miR-15b down-regulated the translation of Wnt4 mRNA

Initially, we investigated the DNA methylation level in the promoter region of *Wnt4* in the TH of F1 mouse brains and the sperm of F0 mice. The promoter region of Wnt4 was enriched in CpGs (SFig. 3A); however, these sites were hypo-methylated, and no significant differences were observed between the controls and the nicotine-treated samples (SFig. 3B and C). As we did not detect any change in methylation state of the Wnt4 promoter, we decided to evaluate changes in miRNAs, another form of epigenetic regulation.

**Figure 5:**
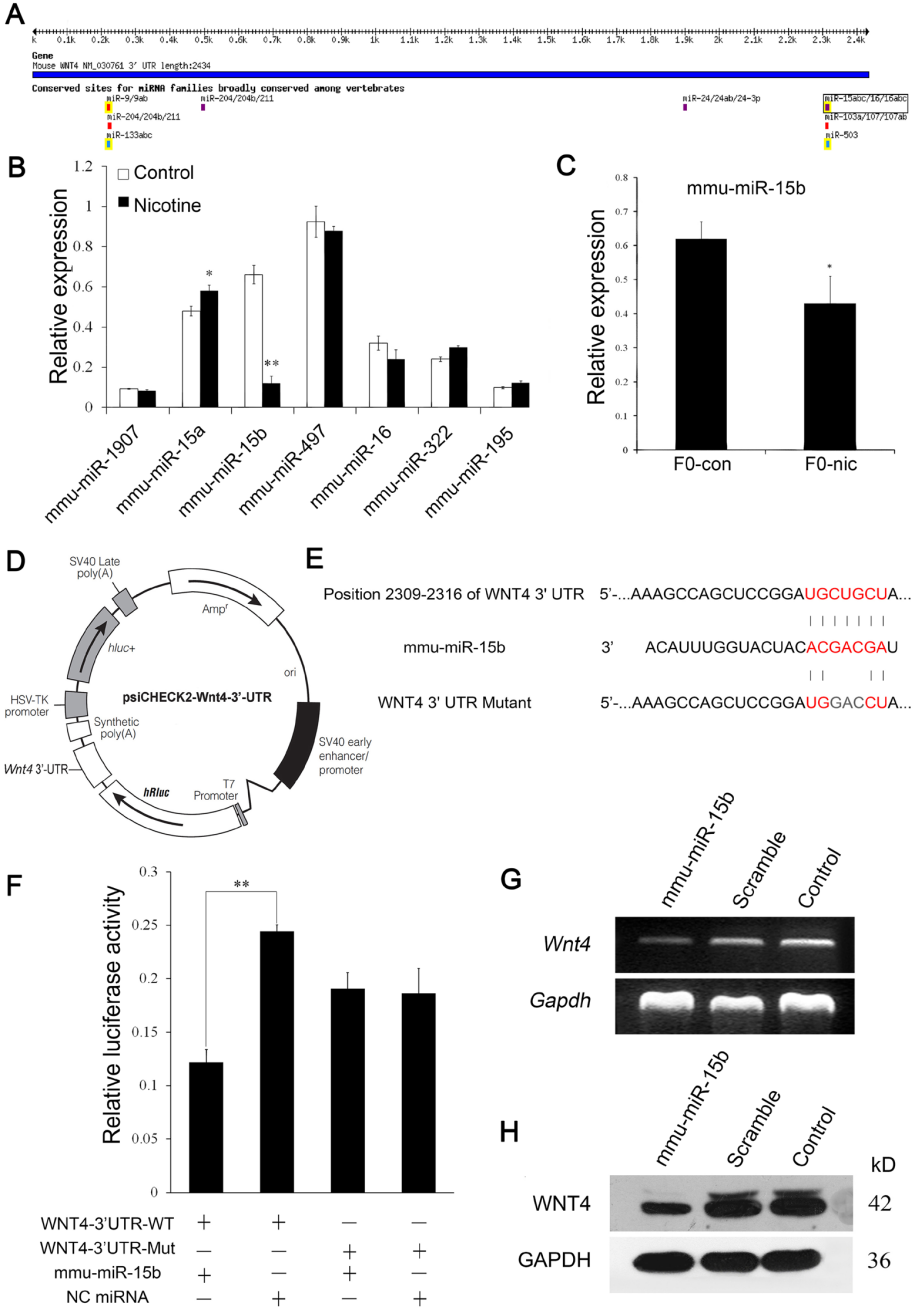
mmu-miR-15b down-regulates the translation of Wnt4 mRNA. (**A**) Diagrammatic sketch of the 3′-UTR region of the murine *Wnt4* gene. The miR-16 microRNA family, including miR-15a, miR-15b, miR-16, miR-1907, miR-497, miR-322 and miR-185, were predicted to bind the *Wnt4* 3′-UTR region. (**B**) Real-time PCR results for the expression levels of these miRNAs in F1-brain tissue. (**C**) Real-time PCR analysis of the mmu-miR-15b in the spermatozoa of F0 mice (n = 4). (**D**) The plasmid profile of the constructed dual-luciferase reporter vector psiCHECK-2-Wnt4 3′-UTR. (**E**) The nucleotide sequences of mmu-miR-15b and the complementary region of the *Wnt4* 3′-UTR. The mutant *Wnt4* 3′-UTR was constructed via point mutations of 3 nucleotides in the middle of the complementary region. The red letters illustrate the sequence alignment of mmu-miR-15b, the Wnt4 3′-UTR and the mutant Wnt4 3′-UTR. (**F**) Luciferase activity reflecting WNT4 expression in HeLa cells was suppressed by mmu-miR-15b, but not NC miRNA. The inhibitory action of mmu-miR-15b was abrogated when the target sites of the *Wnt4* 3′-UTR were mutated (n = 5). (**G**) Western blot results for WNT4 following transfection of mmu-miR-15b into TM3 cells. (**H**) Reverse-transcription PCR results for *Wnt4* after the transfection of mmu-miR-15b into TM3 cells.

The TargetScan database revealed 7 miRNAs as the most likely candidates (Fig. 5A) that might regulate Wnt4 expression levels. Real-time PCR was performed to investigate the expression of mmu-miR-1907, mmu-miR-15a, mmu-miR-15b, mmu-miR-497, mmu-miR-16, mmu-miR-322 and mmu-miR-195 in the brain tissue of F1 mice from the two groups. The results indicated that only mmu-miR-15b was significantly down-regulated after nicotine exposure (Fig. 5B, P = 0.001). Moreover, we used PCR to analyze the expression of mmu-miR-15b in the spermatozoa of F0 mice and obtained results similar to those obtained from the F1 brains. Specifically, nicotine treatment significantly attenuated mmu-miR-15b expression (P = 0.028) in F0 mice spermatozoa (Fig. 5C). To determine whether mmu-miR-15b binds the 3′-UTR of Wnt4 mRNA and down-regulates Wnt4 expression, we cloned the 3′-UTR of Wnt4 into the psiCHECK-2 luciferase reporter vector, and a mutant of the 3′-UTR reporter with the mutation of 3 nucleotides was also constructed (Fig. 5D and E) and the dual-luciferase reporter assay was performed in HeLa cells. As shown in Fig. 5F, the expression of mmu-miR-15b significantly inhibited luciferase activity (P = 0.002), and the ability to inhibit reporter activity was lost in the mutant construct. We further transfected the mmu-miR-15b mimic into the TM3 cell line, which constitutively expresses WNT4. As shown in Fig. 5G and H, the Wnt4 expression in TM3 cells transfected with the mmu-miR-15b mimic was attenuated at both the mRNA and protein levels.

### Nicotine induced DNA hyper-methylation in the CpG island shore region of *mmu-miR-15b* in F1 mouse brains and F0 mouse spermatozoa

**Figure 6:**
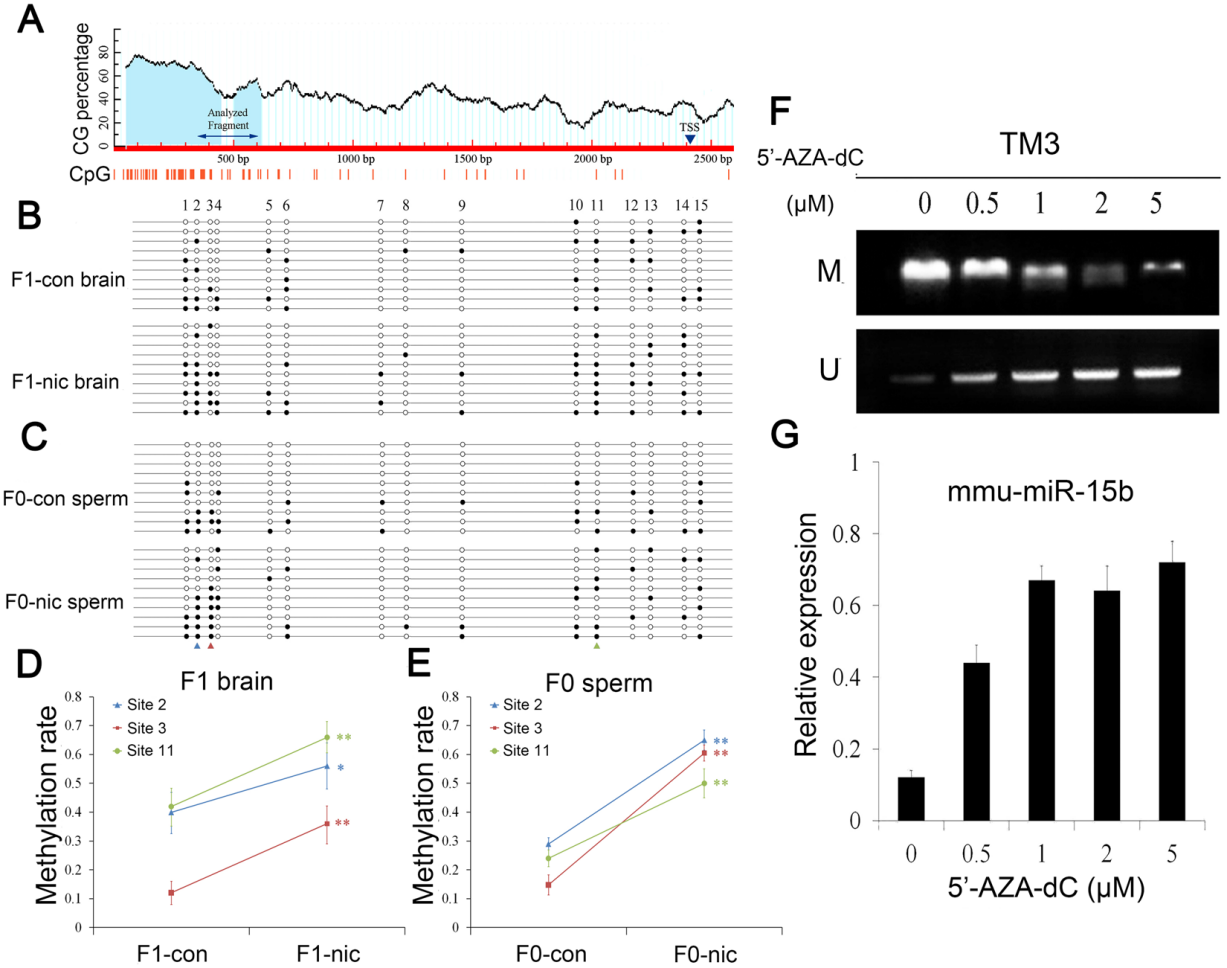
DNA methylation analysis within the CpG shore region of the murine *mmu-miR-15b* gene. (**A**) The predicted CpG island and bisulfite-sequencing PCR primers were designed using the Methprimer tool. The CpG islands are indicated with gray backgrounds, and the BSP primers flanked a 303-bp PCR product upstream of the transcription start position (TTS) of *mmu-miR-15b*. (**B**) The DNA methylation status of the CpG shore region of *mmu-miR-15b* in the thalami of F1 mice from the control and paternal nicotine-exposure groups. Each spot indicates one methylation site (CpG); the black spots indicate methylated cytosines, and the white spots indicate unmethylated cytosines. (**C**) The DNA methylation status of the CpG shore region of *mmu-miR-15b* in the spermatozoa of F0 mice from the control and nicotine-treated groups. (**D**) A histogram of the DNA methylation ratios of the analyzed CpG sites 2, 3 and 11 in the thalami of F1 mice from the control and paternal nicotine-exposure groups (n = 3). (**E**) A histogram of the DNA methylation ratios of the analyzed CpG sites 2, 3 and 11 in the spermatozoa of F0 mice from the control and nicotine-treated groups (n = 3). (**F**) Verification of epigenetic regulation in the mouse TM3 cell line. The panels show the MSP results for the primers designed for the methylated and unmethylated sites in TM3 cells treated with a gradient of 5′-aza-dC concentrations. (**G**) The histogram shows the results of real-time PCR analyses of mmu-miR-15b expression levels in TM3 cells treated with 5′-aza-dC (n = 3).

To further examine whether an epigenetic element of *mmu-miR-15b* is involved in paternal imprinting, we performed DNA methylation analyses of the promoter region of the *mmu-miR-15b* gene. The promoter region of *mmu-miR-15b* lacks CGIs; however, the enrichment of CpG sites in the CGI shore region (Fig. 6A) indicated that *mmu-miR-15b* might be an epigenetically modified gene. Furthermore, we investigated the DNA methylation patterns of the CGIs using primers flanking a 303-bp fragment for bisulfite sequencing analysis. Figure 6B and C illustrate the DNA methylation patterns of the F1-brain and F0-sperm, respectively. The BSP results indicated that the DNA methylation levels of *mmu-miR-15b* in the CGI shore region were significantly elevated at sites 2, 3 and 11 in the F1-brain and F0-sperm after nicotine treatment (Fig. 6D and E). Next, a DNA methylation verification test was conducted using TM3 cells and the results confirmed that mmu-miR-15b expression is elevated by 5′-AZA-dC, which attenuated the methylation levels in the CGI shore region of *mmu-miR-15b* (Fig. 6F and G).

### Overexpression of mmu-miR-15b recapitulates a depression-like phenotype

**Figure 7:**
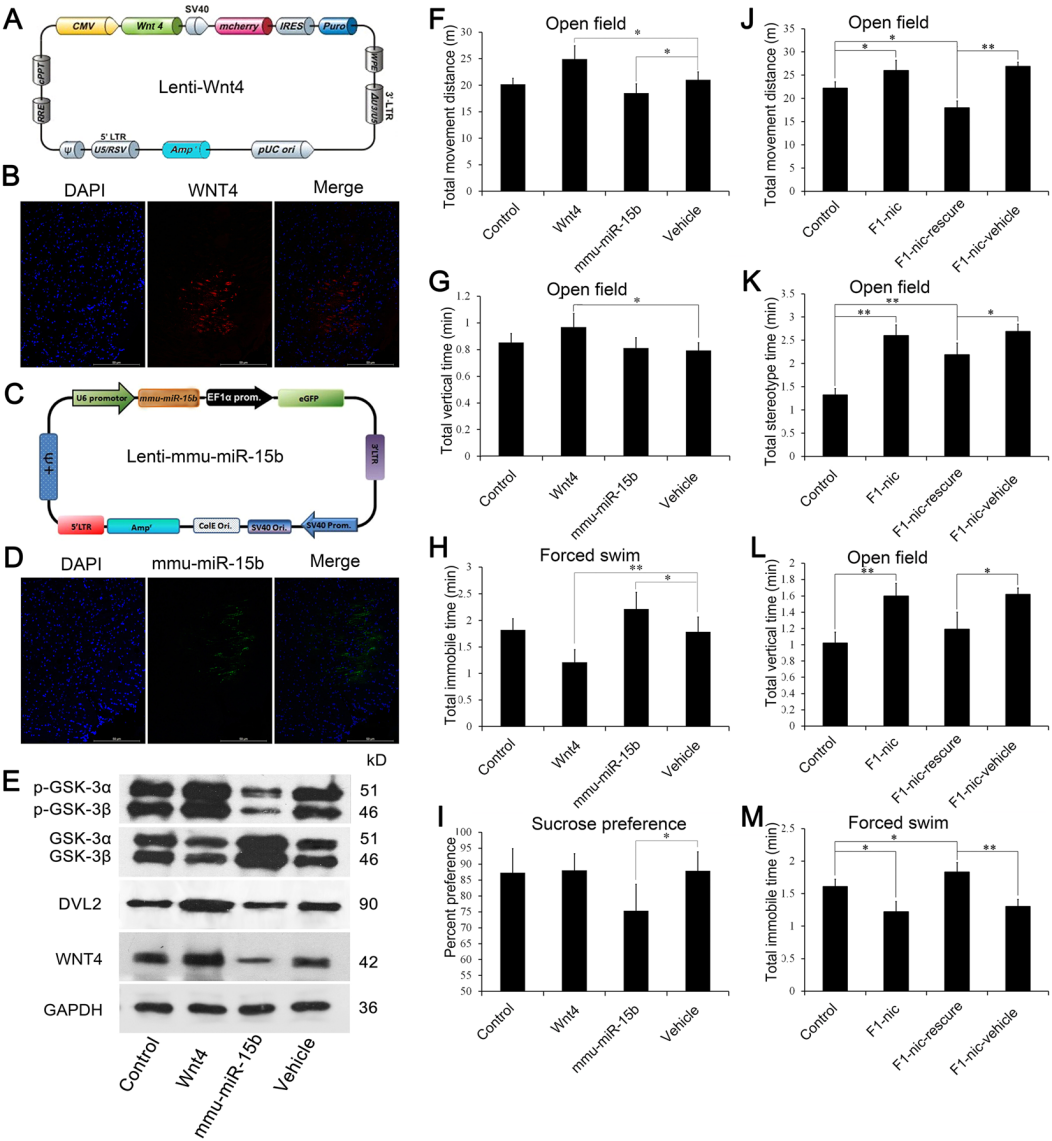
Viral manipulation of mmu-miR-15b and WNT4 in the TH region using brain stereotaxic injection confirmed the neurobehavioral effects of *mmu-miR-15b* and *Wnt4 in vivo*. (**A**) Schematic presentation of the constructed Lenti-Wnt4 vector. (**B**) Representative example of lentivirus-mediated gene transfer in the mouse TH region. The red signal in the fluorescence micrograph shows the viral manipulation of WNT4. (**C**) Schematic presentation of the constructed Lenti-mmu-miR-15b vector. (**D**) Representation of lentivirus-mediated gene transfer in the mouse TH region. The green signal in the fluorescence micrograph shows the viral manipulation of mmu-miR-15b. (**E**) Western blot results for the key proteins in Wnt4 cascade in the TH region of mice that were subjected to the viral manipulated of WNT4 and mmu-miR-15b. (**F**–**I**) Animal behavioral results of mice subjected to lentivirus-mediated gene transfer in the TH region. The histogram shows the total movement distance of the mice (n = 8–12 for each group). (**J**–**M**) Animal behavioral results of F1-nic mice subjected to lentivirus-mediated overexpression of mmu-miR-15b in the TH region. The F1-nic-15b group were subjected to the viral manipulation of mmu-miR-15b, while the F1-nic-vehicle group received injections of blank lentivirus (n = 8–12 for each group).

Furthermore, we assessed the neurobehavioral effects of mmu-miR-15b and WNT4 *in vivo* through the viral manipulation of mmu-miR-15b and WNT4 in the TH region. A diagram of the construct and representative images of the lentivirus-mediated *Wnt4* gene transfer in the murine thalamus are shown in Fig. 7A and B, and those for mmu-miR-15b are shown in Fig. 7C and D. The expression level of the key proteins in Wnt4 cascade in the thalamus of the mice receiving intra-thalamic injections were measured via Western blot analysis and the results validated the efficiency of the viral manipulation (Fig. 7E). C57/BL6J mice then received bilateral intra-thalamic injections of Lenti-Wnt4 or Lenti-mmu-miR-15b, separately, with the empty vector used as a control. These mice were subsequently subjected to behavioral tests to evaluate their neurobehavioral status. In the open-field test, the total distance moved (Fig. 7F, P = 0.018) and total vertical time (Fig. 7G, P = 0.029) were significantly elevated in the Lenti-Wnt4-injected group, and the total distance moved was reduced in the Lenti-mmu-miR-15b group (Fig. 7F, P = 0.034) compared with the vehicle-only group. In the forced swim test (Fig. 7H), the total time of immobility was significantly lower (P = 0.008) in the Lenti-Wnt4-injected group and higher in the Lenti-mmu-miR-15b group (P = 0.036) compared with the vehicle-only group. In the sucrose preference test, the sucrose preference percentage was significantly decreased in the Lenti-mmu-miR-15b transfected group compared with the vehicle group (Fig. 7I, P = 0.019). The other behavioral tests revealed no significant differences between the experimental and vehicle groups (SFig. 4A–F).

To determine whether the behavioral phenotype of F1-nic mice could be attenuated by viral manipulation, the F1-nic mice were also received lentiviral-mediated overexpression of mmu-miR-15b in the thalamus. After 2 weeks of recovery, these mice were subjected to behavioral tests to evaluate their neurobehavioral status. In the open-field test, the overexpression of mmu-miR-15b in the thalamus down-regulated the total distance moved (Fig. 7J, P = 0.002), total time performing stereotypic behaviors (Fig. 7K, P = 0.023) and total vertical time (Fig. 7L, P = 0.025) in F1-nic mice. After the viral manipulation of mmu-miR-15b, the experimental group exhibited a significantly lower total distance moved (Fig. 7J, P = 0.028) compared with control mice, while the total time of stereotyped behaviors did not return to the control level (Fig. 7K, P = 0.008). For the total vertical time, the experimental group showed no difference compared with controls (Fig. 7L). In the forced swim test, the overexpression of mmu-miR-15b significantly elevated the total time of immobility in F1-nic mice (Fig. 7M, P = 0.004), and this parameter was higher compared with control mice (P = 0.039). Based on the behavioral tests, the viral manipulation of mmu-miR-15b in the thalamus of F1-nic mice partially attenuated the behavioral phenotypes resulting from paternal nicotine exposure.

### The neurobehavioral phenotype of F1-nic mice was not passed down to the F2 generation

To observe whether the neurobehavioral phenotype of F1-nic mice is passed down to the F2 generation, the F2-nic and F2-con mice were generated using cross-fostering method as previously described. The animal behavioral tests were performed and the results showed no significant differences between the two groups (SFig. 5A–J). Subsequently, the potential molecular transmission through the paternal line mentioned above was analyzed. Supplementary Fig. 5K shows the western blot films of WNT4 and DVL2 in the brain tissue of F2 mice from the nicotine-treated and control groups, and there was no significant difference between the two groups (SFig. 5L). Real-time PCR analysis of mmu-miR-15b was also performed, and no significant difference was observed between the two groups in the spermatozoa of F1 mice and in the brain tissue of F2 mice. Furthermore, the DNA methylation status of the CpG island shore region of *mmu-miR-15b* was also measured in the spermatozoa of F1 mice and the TH region in F2 mice, and the methylation rates of 3 specific CpG sites showed no significant differences between the two groups (SFig. 5N).

## Discussion

In present study, daily tobacco smoke exposure induced a depression-like phenotype in the F0 generation, resulting in hyperactivity and activated social behavior in the F1 generation. While daily moderate nicotine exposure replicated the depression-like phenotype in the F0 generation and attenuated the depressive level, resulting in hyperactivity in the F1 generation. Remarkably, the murine model of nicotine exposure recapitulated most behavioral phenotypes in the tobacco exposure model, consistent with human epidemiological studies. It is worth noting that in addition to molecular mechanisms, parental information can also be passed to the offspring via cultural or social inheritance systems^25^. However, maternally provided social inheritance is unlikely in our paternal effect system because of the cross-fostering method. As a consequence, the molecular etiology underlying the behavioral alterations observed in the F1-nic mice are most likely inherited from the epigenetic information in the sperm of the F0 mice. Therefore, the intergenerational effects transmitted via the male germline were the focus of the present study. From the gene transcription profiling of the sperm of F0 mice, we observed that the nicotine-induced genes were primarily enriched in the Wnt4 signaling pathway. The disruption of Wnt signaling adversely affects brain development and has been associated with the pathophysiology of several neurological disorders^26^. Recent studies have suggested important roles for the Wnt signaling pathway in bipolar disorder (BP)^27^ and MDD^28^. Multiple components of the Wnt signaling pathway have been implicated in neurogenesis and neurological anomalies, including Frizzled receptors, Dvl and GSK3β. Dvl and β-catenin play critical roles in axon differentiation and dendritic arborization^29^. Moreover, The expression level of GSK3β has been associated with anxiety-like and depression-like behaviors^30, 31^. In addition to expression levels, GSK3α and GSK3β are regulated by inhibitory phosphorylation on Ser21-GSK3α and Ser9-GSK3β. The inhibitory control of GSK3 is crucial for the normal function of neurons^32^. Deficiencies in the signaling pathways that normally maintain the inhibition of GSK3 could induce the up-regulation of GSK3 activity, thereby promoting susceptibility to depression^33^. In the present study, paternal nicotine exposure induced the up-regulation of the Wnt4 pathway in the thalamus of the F1 mouse brain. WNT4-induced inhibitory phosphorylation of GSK3 might represent the molecular etiology of the behavioral alterations discussed above.

Multiple epigenetic mechanisms have also been implicated in the etiology of multiple psychiatric disorders^34^. The results of the present study indicated that the over-expression of mmu-miR-15b in the mouse brain induced hypoactivity and depression-like behavior. Herein, we validated *Wnt4* as a novel target gene of mmu-miR-15b using a dual-luciferase assay. Mmu-miR-15b is highly conserved relative to human miR-15b. Increases in the cortical expression of miR-15b have previously been associated with schizophrenia^35^ but the underlying mechanism remains unknown. At the molecular level, miR-15b plays an important role in cell proliferation and apoptosis by targeting the mRNA of *CCND1* (Cyclin D1) and *BCL2* (*B-cell CLL/lymphoma 2*)^36^. Based on these reported target genes, the other effects of attenuated mmu-miR-15b levels in the mouse brain resulting from paternal nicotine exposure require further study, particularly in terms of neurogenesis. Besides miRNAs, DNA methylation is the most widely studied epigenetic mechanism and tobacco smoking is perhaps the best-studied environmental factor affecting DNA methylation. There are robust correlations between prenatal maternal smoking and DNA methylation patterns in the umbilical cord, placenta, and offspring^37^. In the present study, we focused only the DNA methylation and miRNAs in sperm, but our data do not rule out the possibility of inheritance through other epigenetic mechanisms. The expression level of mmu-mmiR-15b was regulated by DNA methylation in the CGI shore region, and alterations in DNA methylation patterns resulting from nicotine exposure were observed in the spermatozoa of F0 mice and the brains of F1 mice. Additionally, RNA-dependent processes in sperm also contribute to the transmission of acquired traits in mammals^20^, but the precise mechanism remains unknown. We proposed that the alterations of the sncRNA levels in sperm might only be markers or side effects of other epigenetic modifications. In the present study, nicotine elevated the DNA methylation level in the CGI shore of *mmu-miR-15b* in the sperm of F0 mice. Tissue- and disease-specific differentially methylated DNA regions occur more frequently within CGI shores than within CGIs^38^. Such epigenetic modifications are subsequently passed to the next generation and imprint the brain, leading to the induction of neurobiological abnormities. Moreover, the intergenerational effects mediated by mmu-miR-15b were not passed down to the F2 generation. This phenomenon might suggest the reprogramming of DNA methylation during spermatogenesis in F1-nic mice. Evolutionarily, the reprogramming of epigenetic modifications can be considered as a protective mechanism that insulates further generations from the intergenerational effects of environment substances. The precise molecular mechanism underlying the epigenetic regulation of the CGI shore of mmu-miR-15b during spermatogenesis and embryonic development remains elusive.

**Figure 8:**
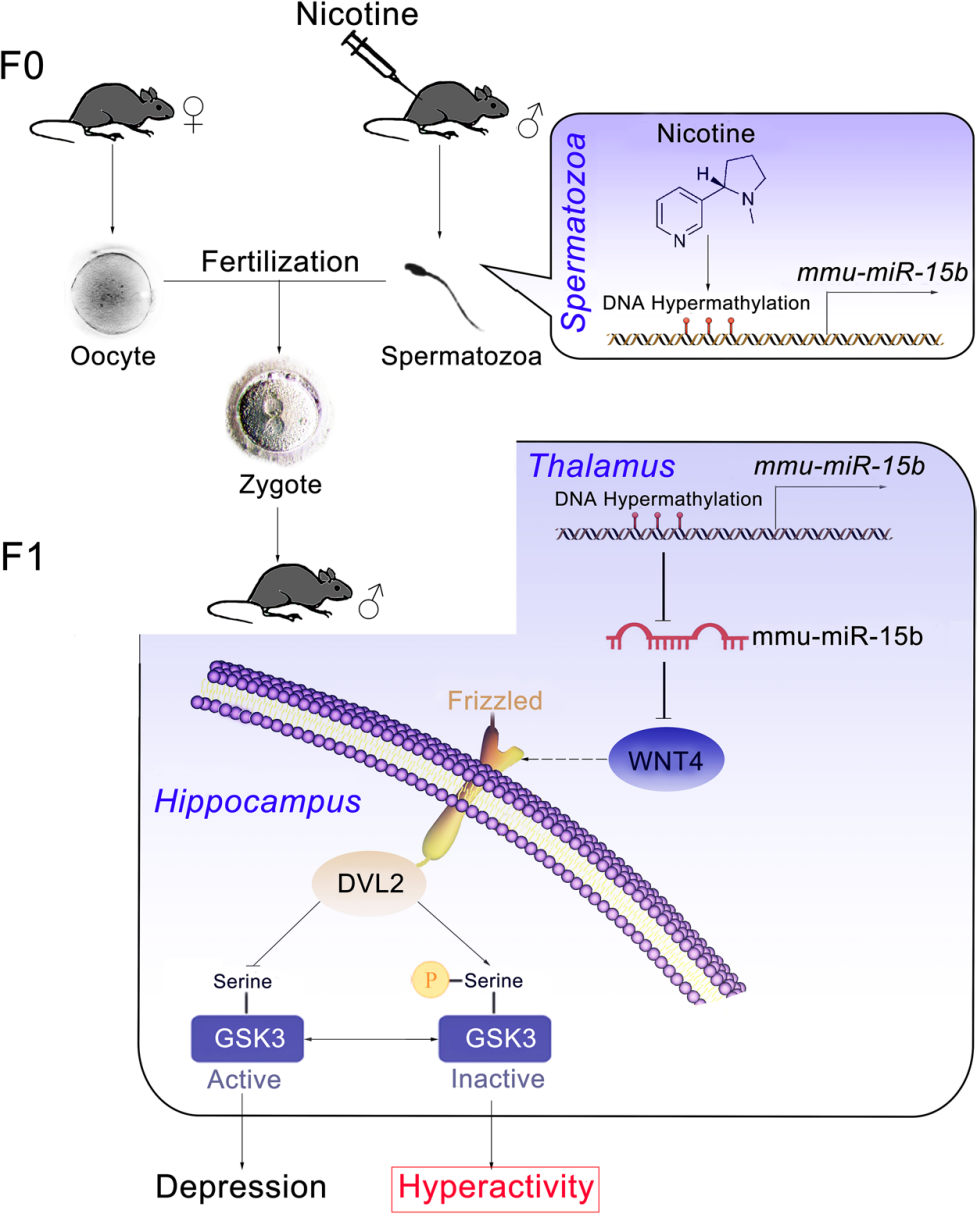
Diagrammatic sketch of the mechanisms underlying paternal nicotine exposure-induced behavioral alterations in the F1 generation.

As summarized in Fig. 8, the present study provides solid evidence regarding the mechanisms underlying paternal nicotine exposure-induced behavioral alterations in F1 generations. Nicotine exposure induces epigenetic mmu-miR-15b downregulation due to CGI shore hyper-methylation in murine spermatozoa, and these epigenetic modifications may mediate the intergenerational inheritance of nicotine-induced neuropsychological disorder. Reduced mmu-miR-15b further elevates the expression of its target gene Wnt4 in the thalamus of F1 brains, followed by the activation of the Wnt4 pathway. The canonical Wnt4 pathway induces inhibitory GSK3 phosphorylation, ultimately inducing hyperactivity and alleviating depression in the offspring.

## Methods

### Animals and Nicotine Treatments

The animal experiments in this study were approved by the bioethics committee of Shanghai Jiao Tong University and all animal studies were performed in accordance with the China State Food and Drug Administration (SFDA) guidelines. Forty 6-week-old male C57BL/6 J mice (acquired from Shanghai SLAC Laboratory Animal Co. Ltd.) were randomly divided into control and nicotine-treated groups containing 20 mice each. The nicotine-treated group (F0-nic) received total 0.2 mg/100 g free-base nicotine via intraperitoneal injection to mimic the blood plasma levels of nicotine in heavy smokers (≥20 cigarettes/day)^39^. The mice received nicotine treatment 4 times per day (q.q.h. in daytime); thus, a lower dose (0.05 mg/100 g) was received in each injection to avoid malaise or sickness. The control group (F0-con) received a daily equivalent of saline. After 5 weeks of treatment, the F0-con and F0-nic mice were mated with normal female C57BL/6 J mice to generate the F1 offspring, designated as the F1-con and F1-nic groups, respectively. The male mice continued to receive nicotine treatment during mating (5 days) until the copulation plugs were observed. Subsequently, the male mice were removed from dam after mating to prevent any exposure to the pups. In the absence of nicotine exposure, when F1 mice were sexually mature, 40 F1 mice from each of the F1-nic and F1-con groups were randomly selected to perform behavioral tests. The mice were group housed with free access to food and water and maintained on a 12:12 h light: dark cycle. For the tobacco smoke-treated mice (F0-smo), an apparatus was designed to expose mice to tobacco smoke, mimicking the exposure of heavy smokers^40^. Specifically, a 150 W vacuum pump was used to generate the suction to draw cigarette smoke into a 30 cm × 30 cm glass box. After the mice were placed in the box, the vacuum pump was concurrently turned on to draw cigarette smoke into the box. After depleting the cigarette, the vacuum pump was turned off and the mice continued to be exposed to the smoke for 1 h. On each occasion, 20 mice were exposed, and the mice were exposed twice a day at an interval of 1 h. The mice in the control group (F0-nos) were placed in a box under identical conditions, but no cigarette was lit. After 5 weeks of treatment, the F0-smo and F0-nos mice were also mated with normal female mice to generate F1 offspring (F1-smo and F1-nos) for further behavioral analysis. All the F1 mice were cross fostered according to the standard protocol.

### Open field Test

In the open field task, each mouse was gently placed into the center of an open field (27.5 cm × 27.5 cm × 25 cm) and remained in the box for a period of 20 minutes. Exploration and arousal were measured as the number of grids crossed and reared using a photo beam activity system. The total movement distance, total stereotype time, total vertical time, time in the central zone and total jump counts were measured.

### Elevated Plus Maze

The elevated plus maze tests were performed in an apparatus comprising four arms (5 cm × 5 cm × 30 cm): two oppositely positioned arms were closed with opaque walls, and the other two arms were open. All of the arms were interconnected via a 5 cm × 5 cm platform, and the apparatus was elevated at 60 cm above the ground. Individual mice were placed at the distal end of one open arm facing away and released. The total time in the open region was measured and analyzed.

### Novel Object Recognition

During training, the mice were placed in the experimental box and exposed to two identical objects for 10 min. After 24 h, the mice were placed back into the same box for the NOR test. To avoid bias resulting from general object preference, the two objects (A and B) used during training and testing were counterbalanced, such that half of the mice in each group were trained with object A and tested with object B and half of the mice in each group were trained with object B and tested with object A. The times that the mice spent with old and novel objects were analyzed.

### Social Chamber Test

The mice were placed in a clear acrylic box partitioned into three chambers. The corners of the two side chambers housed empty black wire-mesh cylinders. The mice were examined under two conditions. Under the first condition, the mice were placed in the center chamber. The partitions were removed, and the animal was permitted to freely explore the chambers. The times and frequencies the animals spent in each of the three chambers and around the cylinders were recorded. The mice were subsequently placed back in the center chamber after 10 min. In the second condition, an unfamiliar C57BL/6J mouse was placed in one cylinder. The test mice were subsequently permitted to explore the chamber and the times and frequencies spent in the three chambers and at the cylinder were recorded.

### Forced Swim Test

The mice were forced to swim for a 5-min period in a vessel containing 40-cm-deep water maintained at 25 ± 1 °C. The time taken to reach the first floating state (i.e., an immobile state with only small limb movements to remain afloat) and the total time spent in an immobile state were measured and analyzed.

### Sucrose Preference Test

The animals were administered two identical 50-ml bottles with sipper tubes containing water for 2 days. On the third day, the animals were provided with one bottle of drinking water, and the other bottle contained 1% sucrose. The amounts of water and sucrose consumed were measured each day over the next 4 days and recorded as the percentage of sucrose consumed.

### RNA Sequencing and Subsequent Bioinformatics Analysis

After an adult male mouse was euthanized, the cauda of the epididymis was carefully isolated, and the spermatozoa were forced out after a nick was cut in the cauda epididymis. The isolated spermatozoa were suspended in human tube for subsequent treatments. The spermatozoa suspended in HTF were purified through the swim-up method in Earles balanced salt containing 10% fetal bovine serum and then washed with hypotonic solution containing 0.5% Triton X-100 and 0.1% SDS to lyse the remaining somatic cells and immature sperm. The samples for RNA extraction were ultimately suspended in TRIzol reagent, and the total RNAs were extracted following the manufacturer’s instructions. Approximately 20 μg of purified RNA submitted for sequencing. Following reverse transcription with random primers, the samples were segmented, and the adaptors were added. A TruSeq^TM^ DNA Sample Prep Kit-Set A (Illumina) was used to construct the library. The samples from 3 mice in each group were subsequently sequenced using an Illumina GAIIx Genome Analyzer with 100 cycles and paired-end sequencing. Using the fastx package, clean reads were obtained from the raw data after filtering out the reads containing more than 5% unknown nucleotides and the low-quality reads. “TOPHAT” was used after the reads and junction reads were mapped to the reference sequence. The data concerning gene expression levels estimated from the RPKM values were obtained using “Cufflinks.” All of the data analysis protocols for the sequenced segments were performed according to commonly used methods that have previously been reported^41^. The Blast2GO comprehensive bioinformatics tool was used for functional annotation and ontology analyses of the gene and protein sequences and gene ontology analyses. Genomatix (http://www.genomatix.com) was used for functional analyses of the target genes. The pathway analyses were performed using the KEGG database (http://www.genome.jp/kegg). Web Gestalt (http://bioinfo.vanderbilt.edu/gotm) was used to analyze the phenotypes of the sequenced mRNAs.

### Cell Culture and Treatments

The HeLa cell line was cultured in DMEM medium supplemented with 50 IU/ml penicillin, 50 IU/ml streptomycin and 10% FBS under 5% CO_2_ at 37 °C, and the TM3 mouse Leydig cell line was cultured in DMEM-F12 medium. After the TM3 cells were cultured to approximately 90% confluence, 5-aza-2′-deoxycytidine (5-aza-dC, Sigma, Cat. No. A3656) was added to the culture medium at final concentrations of 0, 0.5, 1 and 2 μM. The cells were harvested using TRIzol (Life Technologies) following 48 h of treatment, and the total mRNA, genomic DNA and protein were obtained according to standard protocols.

### Dual Luciferase Reporter Assay

Luciferase reporters (psiCHECK2-Wnt4-3′-UTR) were constructed by inserting the 3′- UTR fragment of the mouse Wnt4 gene into a psiCHECK2 reporter vector (Promega) using the NotI/XhoI sites. Mutations of the 3′-UTR were generated via the mutation of 3 nucleotides based on the wild-type (WT) construct psiCHECK2- Wnt4-3′-UTR using the Takara MutanBEST Kit (Takara). The mmu-miR-15b mimic was synthesized at Shanghai GenePharma. HeLa cells were cotransfected with a luciferase reporter vector and mmu-miR-15b mimic or scrambled miRNA using Lipofectamine 2000 (Invitrogen). Firefly and Renilla luciferase activities were sequentially measured using dual-luciferase assays (Promega) at 24 h after transfection according to the manufacturer’s instructions.

### Western Blot Analysis

Purified protein samples were isolated using TRIzol reagent and subjected to SDS-polyacrylamide gel electrophoresis. After electrophoresis, the separated proteins were transferred onto PVDF membranes. The PVDF membranes were blocked for 1 h at room temperature and subsequently incubated with diluted primary antibodies at 4 °C overnight. After rinsing 3 times with TBST, the membranes were incubated with secondary antibody (goat anti-rabbit IgG-HRP, at 1:20000 in TBST; Maibio) for 1 h at room temperature. After rinsing, HRP was detected using the Millipore Immobilon Western Chemiluminescent HRP substrate, and the final blot was exposed to X-ray film. The bands were scanned and equilibrated to the protein concentrations of the samples. The following primary antibodies were used for western blotting: GAPDH rabbit mAb (Cell Signaling Technology, Cat No. 2118S), WNT4 rabbit mAb (Sigma, HPA011397), DVL2 rabbit mAb, (Proteintech, 12037-1-AP), GSK3 rabbit mAb (Cell Signaling Technology, 9369S), p-GSK3 rabbit mAb (Cell Signaling Technology, 9327S), and DNMT3A rabbit mAb (Abcam, ab23565).

### Real-time PCR and Reverse Transcription PCR

Purified RNA was isolated using TRIzol reagent (Invitrogen, Carlsbad, CA) and subsequently reverse transcribed into cDNA with a One-Step PrimeScript miRNA cDNA Synthesis Kit (Takara, D350A). Quantitative real-time PCR was performed using Bestar real-time PCR Master Mix (SYBR Green; DBI Bioscience) and an ABI PRISM 7500 system (Applied Biosystems) according to the manufacturer’s instructions. The primers for real-time PCR were designed via Primer-blast (http://www.ncbi.nlm.nih.gov/tools/primer-blast/) and synthesized at Invitrogen. The transcript levels of the target genes were normalized against the inner reference gene U6. The relative quantifications (RQs) were performed using the 2^−Δ(ΔCt)^ method, where RQ or the fold-change is equal to 2^−((Mean ΔCt Target) − (Mean ΔCt Calibrator))^.

### Immunofluorescence (IF)

The PFA-fixed brain tissue of the mice from the two groups were immersed and embedded in paraffin. Subsequently, 5-μm sections were cut and mounted onto poly-L-lysine-coated glass slides. The sections were baked at 85 °C for 15 min and subsequently deparaffinized according to standard protocols. The rehydrated sections were washed, and 0.01 M citrate buffer (pH 6.0) was used for antigen retrieval in a pressure-cooker. After natural cooling in citrate buffer, the sections were treated with 1% Triton X-100 for 15 min and incubated with 5% BSA (in TBS, pH = 7.4) for 30 min, followed by rinsing with TBS (3 × 5 min) and overnight incubation with the primary antibodies at 4 °C. After washing with TBS, the sections were incubated with the indicated FITC-conjugated mouse anti-goat IgG (Proteintech Group, SA00003-2, 1:200 in TBS) at 37 °C for 1 h. After washing three times with TBS, the sections were mounted using DAPI mounting medium. The fluorescence images were recorded with a Leica DM2500 fluorescence microscope.

### Bisulfite Sequencing (BSP) and Methylation-Specific PCR (MSP)

Purified genomic DNA was isolated using the phenol-chloroform extraction method. Next, 1 μg of purified DNA was treated using the Methylamp DNA Modification Kit (Epigentek) according to the manufacturer’s instructions. The eluted DNA (5 μl) was PCR amplified using *mmu-miR-15b*-specific bisulfate sequencing primers (15b-BSP-S: 5′-GAAGTTTGTGGAGATTTTTTGAG-3′; and 15b-BSP-A: 5′-AAAAAACAATCCAACAATAAAAAAT-3′). The PCR product (303 bp) was recovered, purified and cloned into the pMD19-T vector (Takara, Japan). The cloning vector was transformed into competent bacteria, and 20 clones from the nicotine-treated and control samples were sequenced. The sequencing results were analyzed using BiQ analyzer (http://biq-analyzer.bioinf.mpi-inf.mpg.de/). For the methylation-specific polymerase chain reactions (MSPs), genomic DNA was isolated from 5-aza-dC-treated TM3 cells and subsequently treated with sodium bisulfate. Total volumes of 2.5 μl of the treated DNA were amplified with two sets of MSP primers (15b-M-S: 5′-AGGAATTTTATGTAGTTTGTTTTAACG-3′ and 15b-M-A: 5′-ACACCTACACTCATATACATATACCCG-3′; 15b-U-S: 5′-AATTTTATGTAGTTTGTTTTAATGG-3′ and 15b-U-A: 5′-ACACCTACACTCATATACATATACCCAC-3′). For the BSP and MSP, EX-Taq HS (Takara, RR006A) and touchdown PCR with annealing temperatures of 63 °C to 58 °C were used to ensure the generation of more specific PCR products. The 291-bp PCR products were resolved using 2% agarose gel electrophoresis.

### Mouse Brain Stereotaxic Injection

C57BL/6J mice were anesthetized with pentobarbital sodium (8.5 mg/100 g) and placed into a stereotaxic apparatus (David Kopf). The skin was incised along the midline, and the connective tissue was removed with 10% H_2_O_2_. Burr holes were drilled after the bregma was located. A Hamilton syringe needle was lowered, and the vectors were infused bilaterally at a rate of 0.3 μl per minute in total volumes of 1 μl. The coordinates of the needle tip as measured from bregma were as follows: antero-posterior (AP): −1.6 mm; medial-lateral (ML): ±1.0; and dorsal-ventral (DV): −3.2 mm. The needle remained in place for 3 min following the injection to limit suction of the vector up the needle track.

### Statistical Analysis

All statistical analyses were performed with SPSS version 11. The independent experiments were performed at least in triplicate, and all values are expressed as the means ± SD. Student’s t-tests were used to compare the results between the two groups. Significant differences are indicated by “*” (P < 0.05), and extremely significant differences are indicated by “**” (P < 0.01).

### Data Availability

The datasets generated during and/or analyzed during the current study are available from the corresponding author on reasonable request.

## Electronic supplementary material

Supplementary infomation
