# Supplementary material for: Paternal nicotine exposure defines different behavior in subsequent generation via hyper-methylation of mmu-miR-15b
Source: Sci Rep. 2017 Aug 4;7:7286. doi: 10.1038/s41598-017-07920-3 (PMC5544724; doi:10.1038/s41598-017-07920-3)
Supplement: Supplementary file 1 — Supplementary infomation [file 41598_2017_7920_MOESM1_ESM.pdf]

Paternal nicotine exposure defines different behavior in subsequent generation via hyper-methylation of *mmu-miR-15b*

Jingbo Dai[1, 6, 7], Zhaoxia Wang[1,7], Wangjie Xu[1,7], Meixing Zhang[1], Zijue Zhu[1], Xianglong Zhao[1], Dong Zhang[1], Dongsheng Nie[1], Lianyun Wang[1,*] , Zhongdong Qiao[1,2,3,4,5,*]

[1] School of Life Science and Biotechnology, Shanghai Jiao Tong University, 800 Dongchuan Road, Shanghai, 200240, P. R. China

[2] Bio-X Institutes, Key Laboratory for the Genetics of Developmental and Neuropsychiatric Disorders （Ministry of Education）, Shanghai Jiao Tong University, 1954 Huashan Road, Shanghai 200030, China

[3] Brain Science and Technology Research Centre, Shanghai Jiao Tong University, 800 Dongchuan Road, Shanghai 200240, China

[4] Shanghai Key Laboratory of Psychotic Disorders, Shanghai Institute of Mental Health, Shanghai Jiao Tong University, 600 South Wan Ping Road, Shanghai 200030, China

[5] Shanghai Key Laboratory of reproductive medicine, School of medicine, Shanghai Jiao Tong University, 280 South Chongqing Road, Shanghai 200025, China

[6] College of Medicine, University of Illinois at Chicago, 909 S Wolcott Ave, Chicago, IL, 60612.

[7] These authors contributed equally to this work

*Correspondence to: Prof. Dr. Zhongdong Qiao, Prof. Dr. Lianyun Wang

E-mail: zdqiao@sjtu.edu.cn

Tel.: +86 21 34204925

Fax: +86 21 54747330

Supplementary figure 1

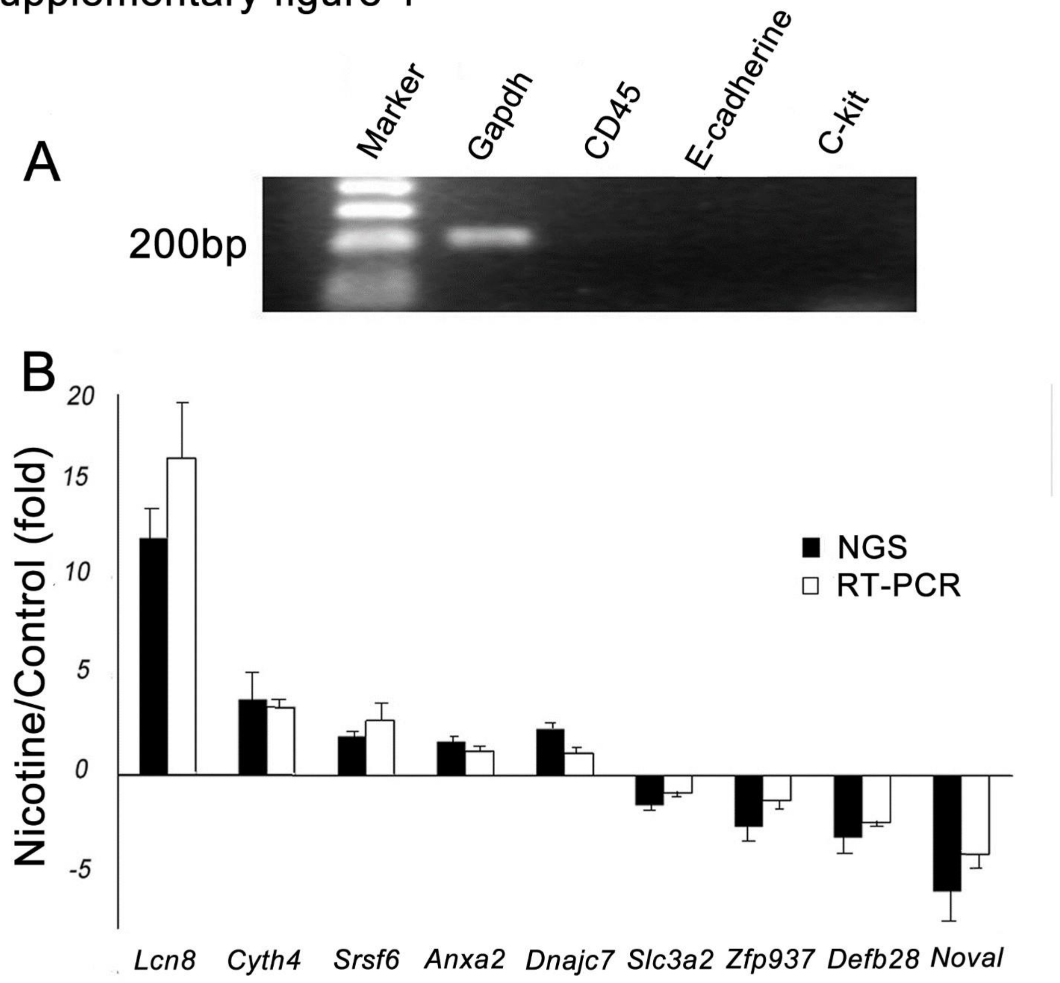

Supplementary figure 1. Verification of the spermatozoal purification and RNA-seq results in murine spermatozoa. (A) RT-PCR results of the three marker genes in murine spermatozoa. (B) Rea-time PCR results of nine differentially expressed genes in the spermatozoa (n=3).

Supplementary Fig 2

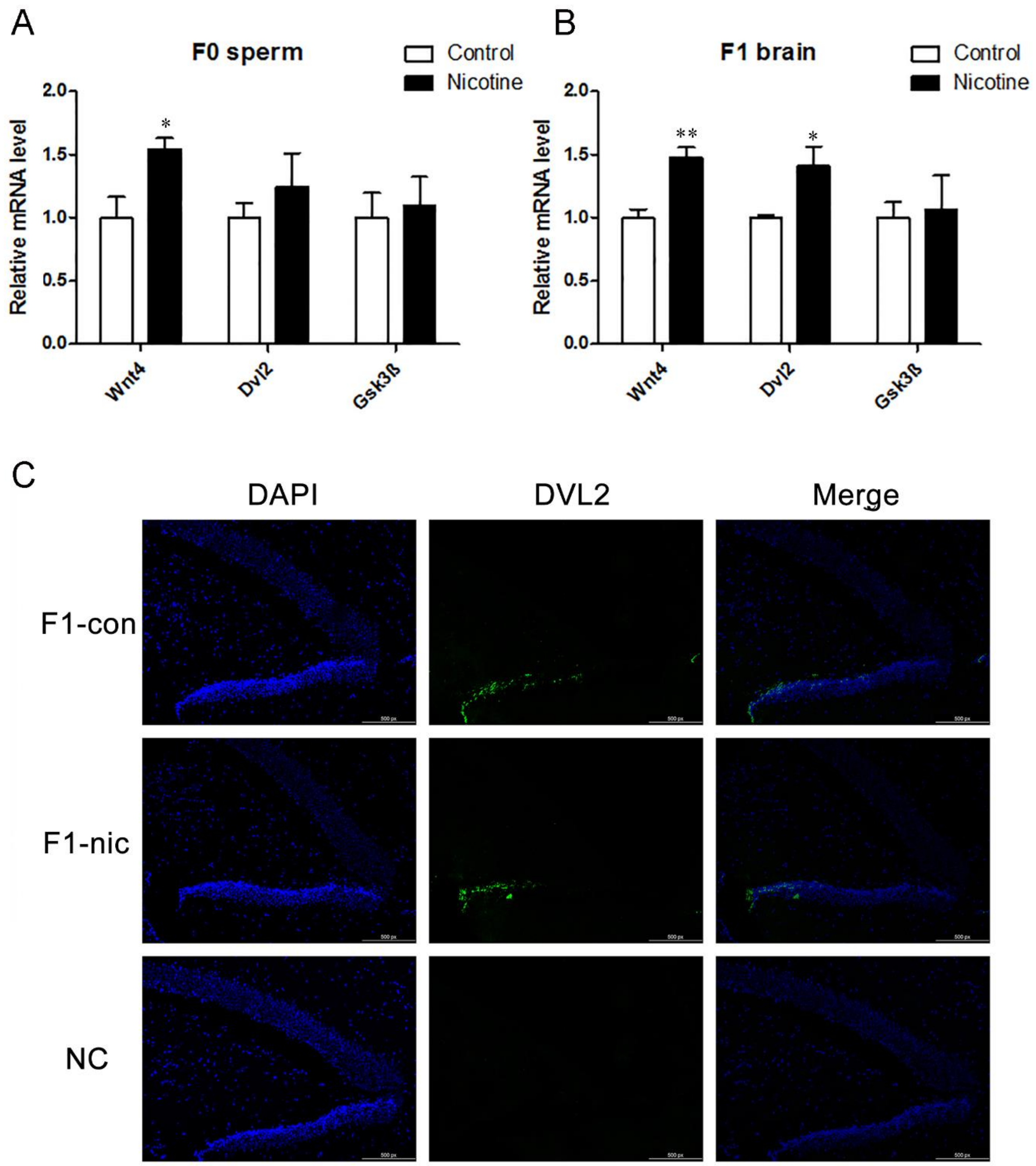

Supplementary figure 2.(A) Rea-time PCR results of *Wnt4, Dvl2* and *Gsk3β* in the sperm of F0 mice. (B) Rea-time PCR results of *Wnt4*, *Dvl2* and *Gsk3β* in the brain tissue of F1 mice. (C) Immunofluorescence staining of DVL2 (green) and DNA (blue) in the DGs of HPF regions in F1-con and F1-nic mice.

Supplementary figure 3

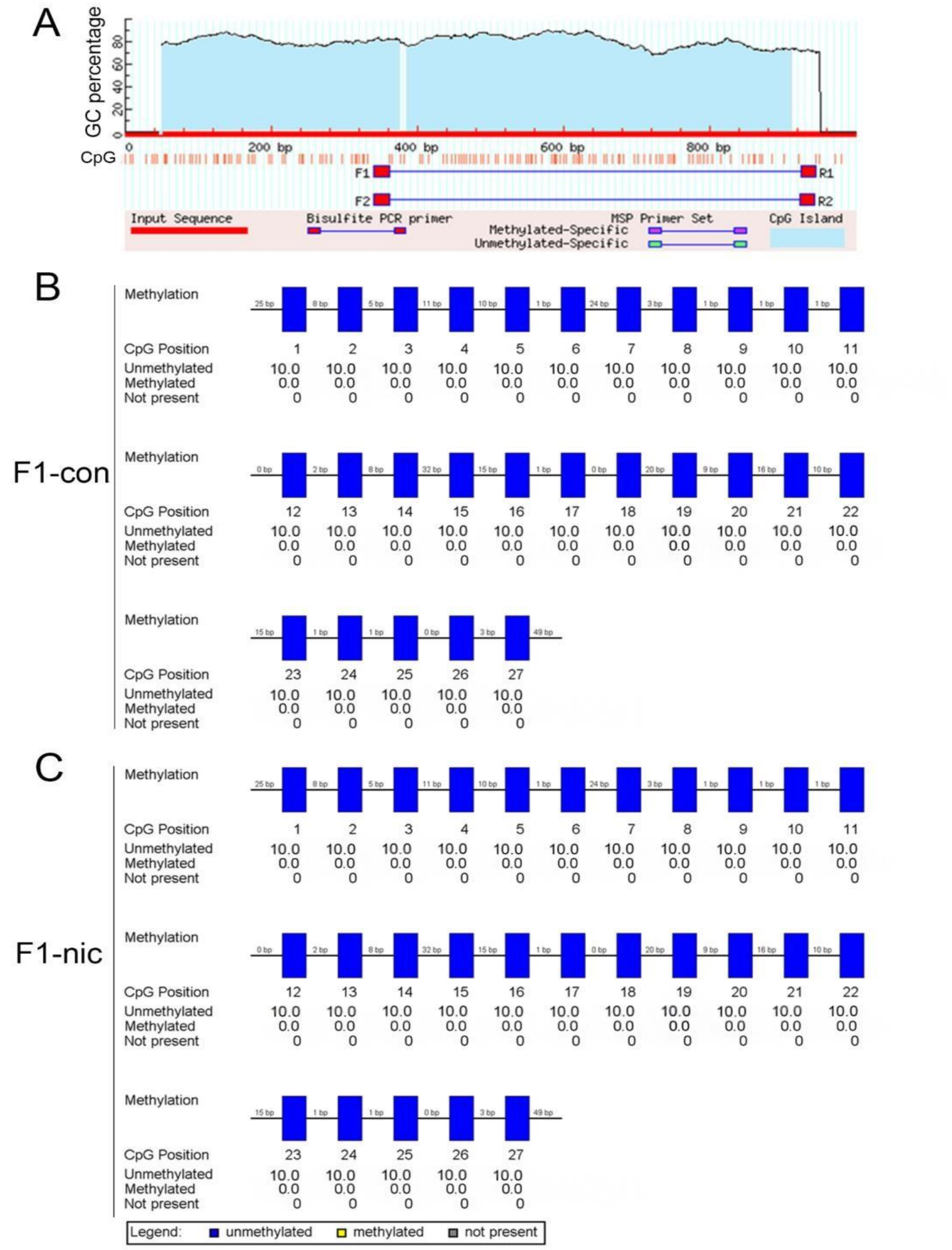

Supplementary figure 3. DNA methylation analysis of the promoter region of the murine *Wnt4* gene. (A) The predicted CpG island and bisulfite-sequencing PCR primers were designed using the Methprimer tool. The CpG islands are indicated with gray backgrounds, and the BSP primers flanked a 303-bp PCR product upstream of the transcription start position (TTS) of mmu-miR-15b. (B) The DNA methylation status of the promoter region of the *Wnt4* gene in the brain tissue of mice from the F1-con group. Each box indicates one methylation site (CpG); the blue indicates methylated cytosines, and yellow indicates unmethylated cytosines. (C) The DNA methylation status of the promoter region of *Wnt4* in the brain tissue of mice from the F1-nic group.

S Fig 4

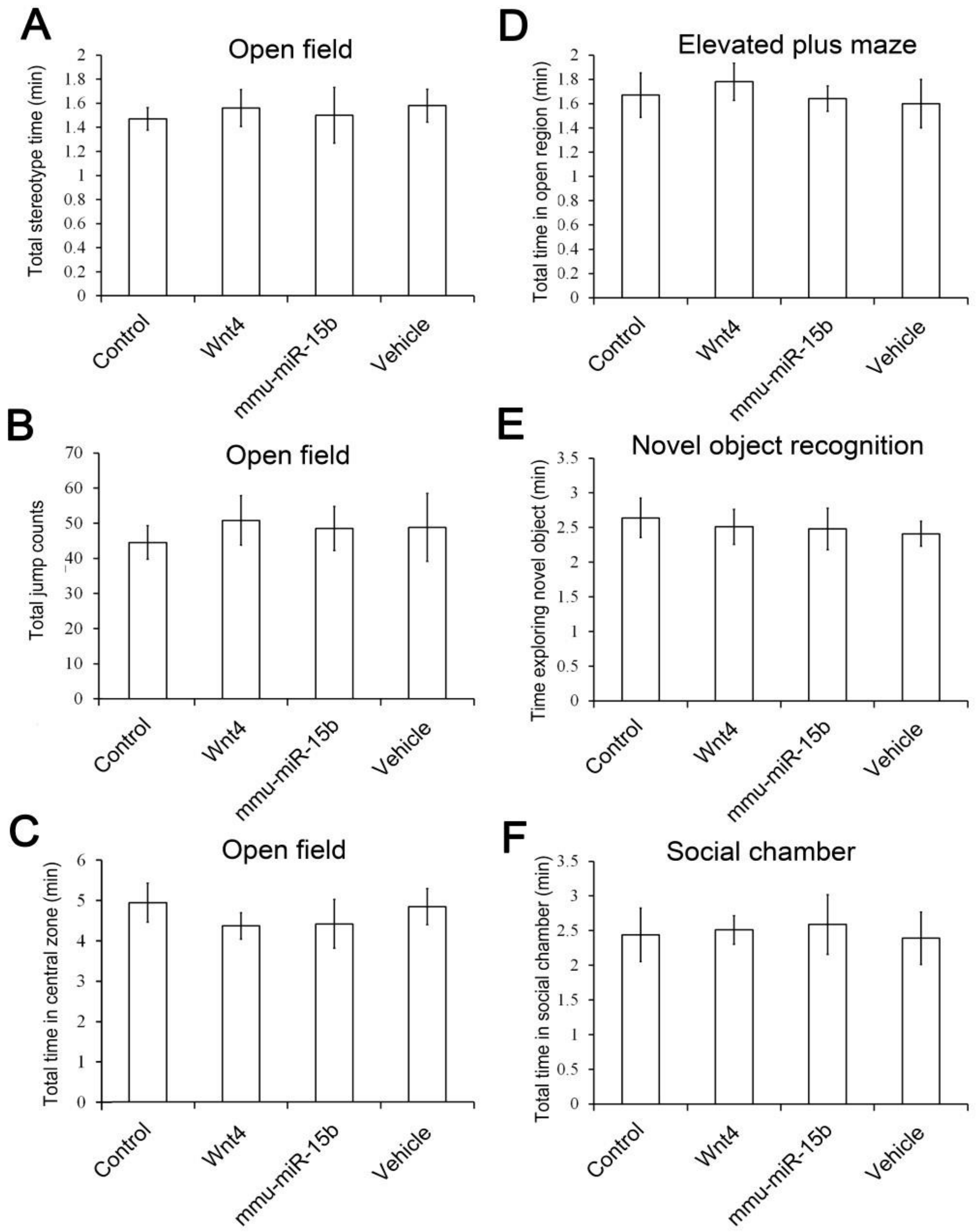

Supplementary figure 4. Behavioral tests of mice subjected to viral manipulation of mmu-miR-15b and WNT4 in the TH region (n=9-10 for each group). (A-C) Open-field test results from mice subjected to lentivirus-mediated gene transfer in the TH region. The histograms show the total stereotype times, total vertical times and total time in the central zone for the mice. (D) Results of the elevated plus maze from mice subjected to lentivirus-mediated gene transfer in the TH region. (E) Results of the novel object recognition test for mice subjected to lentivirus-mediated gene transfer in the TH region. (F) Results of the social chamber test for mice subjected to lentivirus-mediated gene transfer in the TH region.

S Fig 5

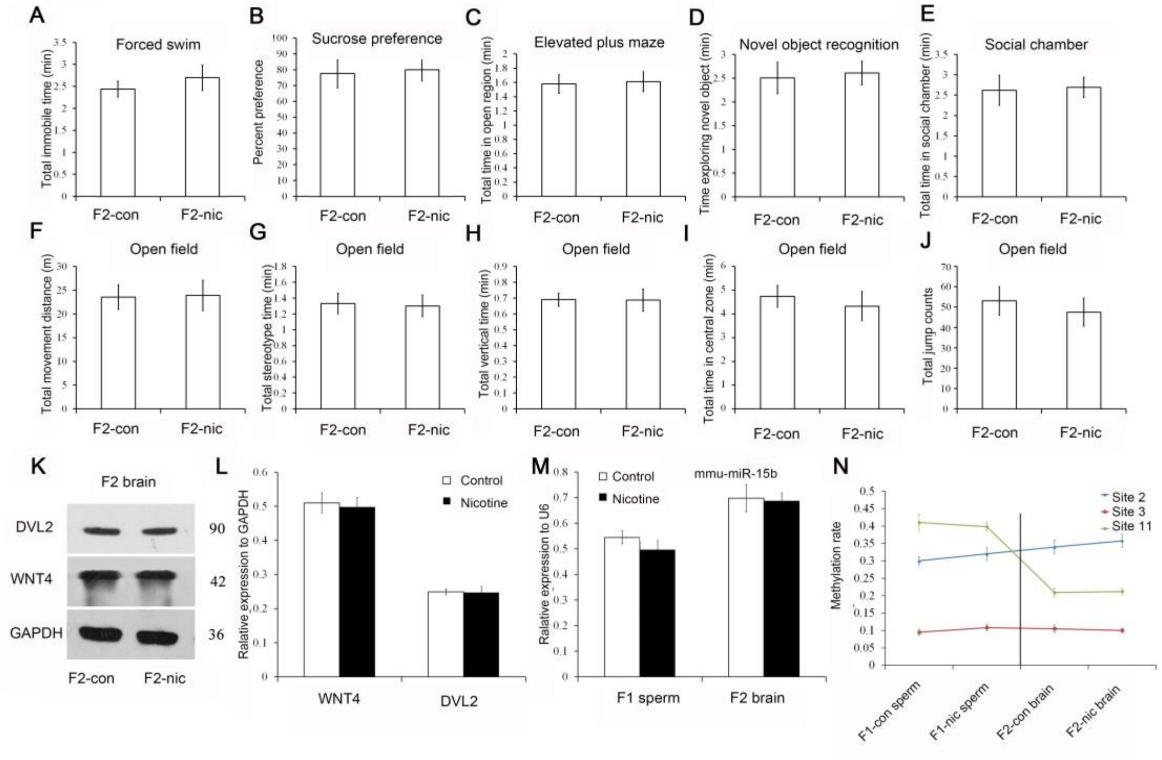

Supplementary figure 5. No significant difference was observed between F2-con and F2-nic mice in the behavioral tests and the molecular traits. (n=10 for each group) (A) Forced swim tests of F2 mice from the nicotine-treated and control groups. (B) Sucrose preference test of F2 mice from the nicotine-treated and control groups. (C) Elevated plus maze test of F2 mice from the nicotine-treated and control groups. (D) Novel object recognition test of F2 mice from the nicotine-treated and control groups. (E) Social chamber test of F2 mice from the nicotine-treated and control groups. (F-J) Open-field test of F2 mice from the nicotine-treated and control groups. (K) Western blot results for WNT4 and DVL2 in the brain tissue of F2 mice from nicotine treated and control groups. (L) Histogram of the western blot results for WNT4 and DVL2 in F2-brain tissue. (M) Real-time PCR results for mmu-miR-15b in the sperm of F1 mice and in the brain tissue of F2 mice. (N) DNA methylation rates of specific CpG sites of *mmu-miR-15b* in the sperm of F1 mice and in the brain tissue of F2 mice.
